# Effect of Basalt Powder Surface Treatments on Mechanical and Processing Properties of Polylactide-Based Composites

**DOI:** 10.3390/ma13235436

**Published:** 2020-11-29

**Authors:** Mateusz Barczewski, Olga Mysiukiewicz, Krzysztof Lewandowski, Daniel Nowak, Danuta Matykiewicz, Jacek Andrzejewski, Katarzyna Skórczewska, Adam Piasecki

**Affiliations:** 1 Institute of Materials Technology, Poznan University of Technology, Piotrowo 3, 61-138 Poznan, Poland; olga.mysiukiewicz@put.poznan.pl (O.M.); daniel.nowak95@wp.pl (D.N.); danuta.matykiewicz@put.poznan.pl (D.M.); jacek.andrzejewski@put.poznan.pl (J.A.); 2 Faculty of Chemical Technology and Engineering, UTP University of Science and Technology, Seminaryjna 3, 85-326 Bydgoszcz, Poland; krzysztof.lewandowski@utp.edu.pl (K.L.); katarzyna.skorczewska@utp.edu.pl (K.S.); 3 Institute of Materials Engineering, Poznan University of Technology, Jana Pawła II 24, 60-965 Poznan, Poland; adam.piasecki@put.poznan.pl

**Keywords:** PLA, poly(lactic acid), composite, basalt, surface modification, mechanical properties

## Abstract

Legislative restrictions and the needs of consumers have created a demand for sustainable materials. Polylactide (PLA) is a biodegradable polyester with advantageous mechanical properties, however, due to its low crystallization rate, it also has low thermomechanical stability. Its range of application temperatures can be widened using nucleating agents and fillers including basalt powder (BP), a waste product from the mining industry. This study analyzed the possibility of enhancing the properties of a PLA-BP composite by chemically treating the filler. Basalt powder was subjected to silanization with 3-aminopropyltriethoxysilane or γ-glycidoxypropyltrimethoxysilane and mixed with PLA at 5–20 wt%. The nucleating effect of a potassium salt of 3,5-bis(methoxycarbonyl) (LAK-301) in the silanized composite was also evaluated. The properties of the materials with silanized BP were compared with the unmodified basalt powder. The miscibility of the filler and the polymer was assessed by oscillatory rheometry. The structure of the composites was studied using scanning electron microscopy and their thermomechanical properties were analyzed using dynamic mechanical thermal analysis. Mechanical properties such as tensile strength, hardness and impact strength, and heat deflection temperature of the materials were also determined. It was concluded that BP-filled nucleated PLA composites presented satisfactory thermomechanical stability without silanization, but chemical treatment could improve the matrix–filler interactions.

## 1. Introduction

Despite the many legislative restrictions and obligations imposed on producers and consumers of plastic products, increasing amounts of polymeric materials end their lifecycle as mixed waste. Mechanical recycling allows for simple and effective management of post-consumer plastics, but in many cases, selective waste collection is not carried out properly, so the waste stream is not recycled [1,2]. Moreover, recycling can delay, but not prevent any existing end-of-life material from reaching final disposal [3,4]. Despite the increasing efficiency of recycling and material collection processes, it is important to develop alternative biodegradable materials, which, in the case of improper disposal, will potentially constitute a lower burden on the natural environment.

Polylactide or poly(lactic acid) (PLA) is a polymer from the group of biodegradable/compostable thermoplastic polyesters and is the compound most widely used as a biopolymer [4,5]. It is mainly used in the food and packaging industries [6]. However, this material has good mechanical and processing properties, which makes it an alternative to petrochemical construction polymers such as polypropylene (PP) or polyoxymethylene (POM). The greatest limitations in its use as a construction material are high brittleness and the glass transition that occurs in the temperature range of applicability of most products (approximately 50 °C). The low thermomechanical stability of PLA results from the low tendency of the polymer to form crystalline structures. This problem can be solved by increasing the amounts of the crystalline phase by introducing heterogeneous nucleants and applying appropriate processing and post-processing conditions as well as using both methods while strengthening the polymer by introducing fillers in the form of powders or fibers.

The production of polymer composites with the use of fillers made of post-production or post-exploitation waste (i.e., the by-products from various industrial processed or post-consumer waste) is a commonly implemented trend, described by scientists and used in industrial practice. Particle-shaped and powder fillers are the simplest to produce by grinding processes. Their application reduces the price of the final product, especially in the case of highly-filled composites [7]. The ground waste of the wooden parts of plants are the most widely described filler in the literature [8,9,10,11,12,13]. The studies have also considered the use of inorganic fillers from the metallurgical, stone, or mining industries [14,15,16,17].

**Figure 1:**
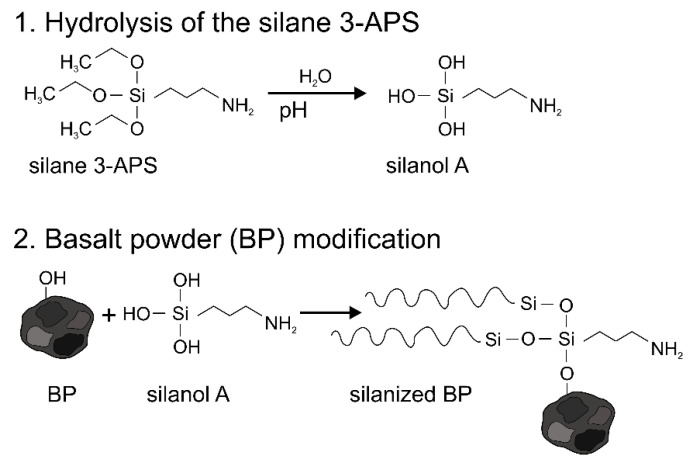
Mechanism of the surface modification of the basalt powder conducted using organosilanes.

Most of the fillers used are characterized by high hydrophilicity, which, considering the hydrophobic nature of most polymers, leads to poor compatibility between them. To increase the interfacial interactions in the composite, various modification methods are used including the introduction of compatibilizers such as maleated coupling agents [18,19,20] or chemical modification of the surface of the fillers. Among the various methods of surface modification of inorganic and organic fillers such as the application of isocyanates [21,22], diazonium salts [23,24], and grafting (e.g., using polyacrylamide [25], or ionic liquids [26,27]), the most commonly-used is silanization [28,29]. By using the appropriate organofunctional silanes, it is possible to achieve the greatest compatibility between the polymer and the modified filler. In the case of using inorganic hydrophilic fillers, the effectiveness of the reinforcement is strongly related to the adhesion at the polymer–filler interface. The use of silanization to modify fillers with a different chemical structure compared to polymers reduces their hydrophilicity and increases their compatibility with almost all types of polymers including thermoset as well as thermoplastic grades [29,30,31,32,33,34,35]. The course of the silanization process has been quite accurately described in the literature and most researchers agree on the defined mechanisms that occur during the attachment of organosilanes to the surface of the filler containing hydroxyl groups. Silane hydrolysis drives silane adhesion to the substrate, resulting in the formation of siloxane bonds on the surface of the substrate. The initial step of hydrolysis may take place in solution or on the surface of the substrate, depending on the amount of water present in the system. Excess water causes polymerization of silanol in the solvent phase, while an insufficient amount of water results in an incomplete single layer [36]. Therefore, it is important that the conditions of the hydrolysis process are carefully selected and controlled by considering the pH as well as the concentration of an aqueous solution [37,38]. The scheme of the mechanism of surface modification of the basalt powder using a silane coupling agent is presented in Figure 1.

So far, the presented results concerning the modification of inorganic particle-shaped fillers has led to the assumption that the application of silane coupling agents is an effective method that improves the final properties of the composites and may suppress the negative effects of the presence of the particle-shaped filler (e.g., effects on the tensile strength or impact resistance). It used to be assumed that the weaker adhesion between rigid particles and polymer led to prevalent debonding at lower stresses. Dike [33,34] described the effect of the silanization of inorganic particle-shaped fillers (barite and huntite–hydromagnesite) using amino-functionalized silane for the properties of the PLA–matrix composites. The results showed that surface treatment improved the stiffness, tensile properties, and strength of the composite-filled silanized particles. In the case of the silanization of organoclays such as montmorillonite (MMT), silanization was not the most effective coupling method; however, additional improvements in the mechanical properties of the composites manufactured with their application result from exfoliation of the layers [39]. In contrast, the mechanism of the toughening of PP using surface-treated glass particles described by Thio et al. showed that weaker interfacial interactions between rigid filler particles and the polymer gave the effect of higher toughness of the composite. This phenomenon relates to the strain dependency of the toughening process induced by the addition of the particle-shaped filler when, in the case of an abrupt impact, the interparticle ligaments are unable to deform plastically [39].

Despite many studies including the application of various modifications of inorganic particles used as a reinforcement of polylactide composites, there is no evidence of the evaluation of the influence of the surface treatment of basalt derivatives in the form of particles. On the other hand, many works contain descriptions of the application of basalt fibers in polylactide composites after being treated using organofunctional silane coupling agents including γ-glycidoxypropyltrimethoxysilane (GPS), 3-aminopropyltriethoxysilane (3-APS), or N-(2-aminoethyl)-3-aminopropyltrimethoxysilane [40,41,42,43]. A common feature of the results presented in all of the mentioned works was the high efficiency of silane interactions, improved interfacial adhesion, and improved strength of composites containing the modified fibers.

Increasing the crystallinity of PLA is one of the most commonly-used methods to improve its stiffness at elevated temperatures. Despite the low ability of PLA to form crystalline structures, the simultaneous use of the appropriate processing conditions with the addition of nucleating agents (NAs) obtains PLA crystallization during non-isothermal cooling during the injection molding process [44]. In the studies reported so far, many different compounds that act as PLA nucleants have been presented including talc [45], aromatic sulfonate derivative (LAK-301) [45,46], phthalhydrazide [47,48], otoric acid [49], and PLA stereocomplexes [50,51]. LAK-301 is one of the most effective NAs, characterized by high availability as well as confirmed effectivity in a wide range of processing conditions [44].

In our former studies, we discussed the possibility of using basalt powder as a replacement for micrometric milled basalt fibers, which can achieve improved thermomechanical properties [52]. It was found that the efficiency of thermomechanical stability increased is a synergic effect of the presence of the basalt derivative and improved crystallinity of the PLA–matrix induced by annealing [52] or by the addition of a heterogeneous nucleating agents [53]. The aim of this study was to justify the use of additional basalt powder in the silane treatment in the simultaneous incorporation of a nucleating agent (LAK-301). The efficiency of two different organosilanes was verified in the course of their influence on the compatibility with the polylactide matrix as well as their impact on the thermomechanical behavior of the composites resulting from the potential additional functionality of the epoxy and amino groups.

## 2. Materials and Methods

### 2.1. Materials and Sample Preparation

A commercial grade of polylactide, namely Ingeo 2500HP by Natureworks (Minnetonka, Mn, USA), was chosen as the matrix of the composites. This material can be characterized by the following properties: melt flow index, MFI of 8 g/10 min (210 °C, 2.16 kg), the density of 1.24 g/cm^3^, and d-isomer content of <0.5%. Natural basalt powder (BP) obtained from the “BAZALT SA” mine in Wilków, Poland was used as the filler. A more in-depth analysis of BP can be found in our previous papers [52,53]. The filler was chemically treated with two types of silane compatibilizers: (3-aminopropyl)triethoxysilane (referred to as 3-APS) and (3-glycidoxypropyl)trimethoxysilane (GPS) supplied by Sigma Aldrich (St. Louis, MO, USA). A potassium salt of 3,5-bis(methoxycarbonyl) under the commercial-grade LAK-301 obtained from Takemoto Oil and Fat Company (Gamagori, Japan) was used as the nucleating agent (further called LAK).

The chemical treatment of basalt powder with the silanes was performed in the following way. First, the compatibilizers (3 wt%. in reference to the amount of used basalt powder) were hydrolyzed in a 4:1 mixture of 96% ethanol (reagent grade, supplied by Chempur, Karlsruhe, Germany) and demineralized water, respectively. This reaction was conducted at room temperature for 1 h. In the case of 3-APS, the pH of the solution was adjusted to 4 using 80% acetic acid (reagent grade, supplied by Chempur, Karlsruhe, Germany). For GPS, the pH was about 7 and did not need adjustment. After hydrolysis, basalt powder was added to the solution for silanization under mechanical mixing for 3 h at room temperature. After that, the modified basalt powder was removed from the mixture, washed several times with demineralized water, and air dried for four days and then at 80 °C for 12 h using a laboratory cabinet drier. The filler treated with 3-APS and GPS are referred to as BA and BG, respectively.

To prepare the composite samples, PLA and the chosen amount of fillers (5, 10, or 20 wt%) and the nucleating agent (1 wt%, in the case of the nucleated series) were dry-mixed and then dried at 50 °C for 24 h in a laboratory cabinet drier Memmert ULE 500 (Schwabach, Germany). Then, the polymer and the filler were mixed in a molten state using a co-rotating twin-screw extruder ZAMAK 16/40 EHD (Skawina, Poland) at 190 °C with a screw speed of 100 rpm. The extrudates were cooled in a forced airflow and pelletized. The normalized specimens for testing were prepared by injection molding using a Battenfeld PLUS 35 machine (Kottingbrunn, Austria). The injection molding was performed in the following conditions: T_melt_ = 190 °C, T_mold_ = 120 °C, p_injection_ = 72 MPa, v_injection_ = 75 mm/s, t_cooling_ = 60 s. Unmodified and nucleated PLA was processed in the same way as its composites.

### 2.2. Methods

The effect of incorporating the organic fillers on the processing properties and changes in the plastification process of the PLA-based composites were evaluated with a torque rheometer. A Brabender plastographometer (Duisburg, Germany) was applied to measure the torque during plastification of PLA and composites filled with basalt powders subjected to surface treatment. The measurements were conducted at 170 °C at a constant rotational speed of 30 rpm in a 50 cm^3^ chamber with an instrument constant K equal to 0.0825 cm^−3^ [54].

Oscillatory rheological investigations were carried using an Anton Paar MCR 301 rotational rheometer (Graz, Austria) with 25 mm diameter parallel plates with a 0.5 mm gap. The experiments were conducted at 200 °C. The strain value applied during frequency sweep experiments was 2% and it was in the range of the linear viscoelastic behavior (LVE) of all samples, which was confirmed during preliminary strain sweep measurements. The angular frequency ω used in the study was in the range of 0.05–500 rad/s. The Rheoplus v.3.40 application was used for fitting the Carreau–Yasuda model to the experimental data.

The scanning electron microscope (SEM), model Vega 5135MM produced by Tescan (Brno, Czech Republic), was used to assess the structure of the composites. The PLA-based composite structures were investigated using a secondary electron signal (SE) with an accelerating voltage of 12 kV. All analyzed samples were broken after being cooled in liquid nitrogen.

The density of the composites was determined by the hydrostatic method using an AXIS AD200 balance (Gdańsk, Poland) and demineralized water as the immersion fluid (*ρ*_*water*, 23°*C*_ = 0.9975 g/cm^3^). The mass of the sample was measured in the air (*m*_1_) and in water (*m*_2_). The density of the sample *ρ* was calculated according to Equation (1):

(1) $$\rho =\frac{m_{1}\cdot \rho_{water,23{}^{\circ}C}}{\left(m_{1}-m_{2}\right)},$$

The mechanical properties of PLA and its composites were determined in the tensile test using a Zwick/Roell 010 universal testing machine (Ulm, Germany). The crosshead speed was set to 1 mm/min during the tensile modulus evaluation and 10 mm/min in the remaining part of the test. Based on the obtained data, values of tensile modulus E, tensile strength Rm, and elongation at break ε were determined. At least five samples of each kind were tested.

The hardness of the composite samples and pure PLA was measured using Brinell’s (ball indentation) method according to the ISO 2039 standard. The measurements were performed on the injection-molded samples using a KB Prüftechnik tester (Hochdorf-Assenheim, Germany). The hardness values of the composites and PLA were determined as the mean value of at least 10 separate measurements.

The impact strength was evaluated using the Dynstat method, in compliance with the DIN 53435 standard, on unnotched samples with dimensions of 4 mm × 10 mm × 15 mm. A Dys-e 8421 apparatus (Leipzig, German Democratic Republic) equipped with a 0.98 J hammer was employed.

Thermal properties of the studied materials such as melting, crystallization, and cold crystallization temperature (T_M_, T_CR_, and T_CC_, respectively) as well as the crystallinity degree were analyzed using the differential scanning calorimetry (DSC) method. Samples of 5 mg were placed in aluminum crucibles with pierced lids and heated from 25 °C to 210 °C with a rate of 10 °C/min, held at this temperature for 10 min, and then cooled back to 25 °C at a rate of 10 °C/min. As the sampler prepared in the same way had a similar thermal history, the information from the first heating/cooling cycle was used in the analysis. A Netzsch DSC 204F1 Phoenix apparatus (Selb, Germany) and a nitrogen atmosphere were used. The crystallinity degree (*X_CR_*) was calculated according to Equation (2):

(2) $$X_{CR}=\frac{\Delta H_{M}-\Delta H_{CC}}{\left(1-\varphi \right)\cdot \Delta H_{PLA100\%}}\cdot 100\%$$

where Δ*H_M_* is the melting enthalpy of the sample; Δ*H_CC_* is the cold crystallization enthalpy of the sample; Δ*H_PLA_*_100%_ is the melting enthalpy of a 100% crystalline PLA; Δ*H_PLA_*_100%_ = 93.7 J/g [55]; and *φ* is the filler content.

The thermogravimetric analysis (TGA) method was employed to study the thermal degradation of the composites and PLA. Samples of 10 mg placed in Al_2_O_3_ crucibles were heated in the temperature range of 30–900 °C at a rate of 10 °C/min using a Netzsch TG209 F1 apparatus (Selb, Germany). An inert atmosphere of nitrogen was applied. Based on the obtained mass vs. temperature curves, its first derivative (dTG) was calculated. Values of 5% mass loss temperature(T_5_) and maximum thermal degradation intensity temperature (dTG peak, T_DEG_) were determined.

Thermomechanical properties of the basalt powder-filled samples and PLA were evaluated by dynamic mechanical thermal analysis (DMTA) using an Anton Paar MCR 301 apparatus operating in torsion mode. The samples were heated from 25 °C to 120 °C at a rate of 2 °C/min. A strain of 0.01% was applied at a frequency of 1 Hz. Based on the obtained data values of storage modulus (G′) in the glassy and rubbery state (30 °C and 80 °C, respectively), glass transition temperature (T_G_, understood as the peak of tanδ vs. temperature curve) and tanδ at T_G_ were determined. Brittleness B was calculated according to Equation (3) as proposed by Brostow et al. [56]:

(3) $$B=\frac{1}{{G^{\prime }}_{30{}^{\circ}C}\cdot \varepsilon }$$

The effectiveness of the filler C was calculated according to Equation (4) [57]:

(4) $$C=\frac{{\left({G^{\prime }}_{30{}^{\circ}C}/{G^{\prime }}_{80{}^{\circ}C}\right)}_{\mathrm{composite}}}{{\left({G^{\prime }}_{30{}^{\circ}C}/{G^{\prime }}_{80{}^{\circ}C}\right)}_{\mathrm{matrix}}}$$

The reinforcement efficiency of the filler r was determined using Equation (5) [58]:

(5) $$r=\frac{\left({G^{\prime }}_{c}/{G^{\prime }}_{m}\right)-1}{V_{f}}$$

where G′_c_ is the storage modulus of the composite; G′_m_ is the storage modulus of the matrix; and V_f_ is the volume fraction of the filler.

The heat deflection temperature (HDT) was evaluated according to the standard ISO 75, method B, using a Ceast HDT Vicat Tester HV3. A heating rate of 120 °C/h and a stress of 0.45 MPa were applied.

## 3. Results

### 3.1. Processing and Rheological Behavior

**Figure 2:**
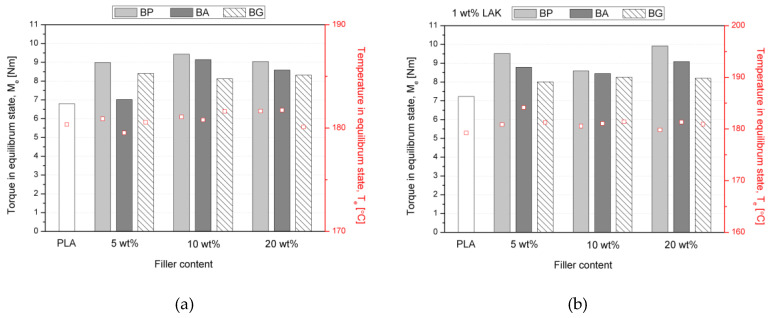
Results of torque rheometry: (**a**) torque (M_e_) at the equilibrium state of the polylactide (PLA) and PLA-composites samples and (**b**) temperature (T_e_) at the equilibrium state of the polylactide (PLA) and PLA-composites samples.

Processing properties of PLA composites filled with various amounts of basalt powder with and without chemical treatment were investigated using torque rheometry. The torque measured in the equilibrium state (M_e_) as well as the temperature value at the end of the measurement (T_e_) are shown in Figure 2. All composites showed increased M_e_ in comparison to unfilled PLA. For all composite series, there was a lack of visible changes in the torque induced by changing the filler content, which can be treated as a beneficial effect because increasing the content of the filler does not suppress the processability of the composites. It can also be seen that surface modification of the basalt filler provided changes in the measured torque. The highest M_e_ values were measured for composites filled with BP and the lowest for BG. The phenomena of limited negative influence of BP addition on PLA-based composites processability was observed and is in line with our earlier studies concerning modification of isotactic polypropylene (iPP) with BP [59]. It was found that the incorporation of basalt powder may improve the processability of iPP by simultaneously reducing the viscosity of the molten composite bulk and increasing the wall-slip effects. For the mentioned iPP-BP composites the torque was comparable for pure polymer and composites, while PLA-based composites revealed limited effects of the BP presence. It should be noted that despite lower modification efficiency of BP on PLA than in the case of polyolefins, the increase of M_e_ was insignificant and suggests a lack of potential limitations during technological processes in the molten state. The temperature measured at equilibrium was similar for all compositions and comparable with neat PLA. Contrary to results presented by Du et al. [60], the addition of modified filler did not change the T_e_ in comparison to composites containing BP, which suggests a lack of a significant interfacial bonding resulting from the high reactivity of the filler on the PLA.

**Figure 3:**
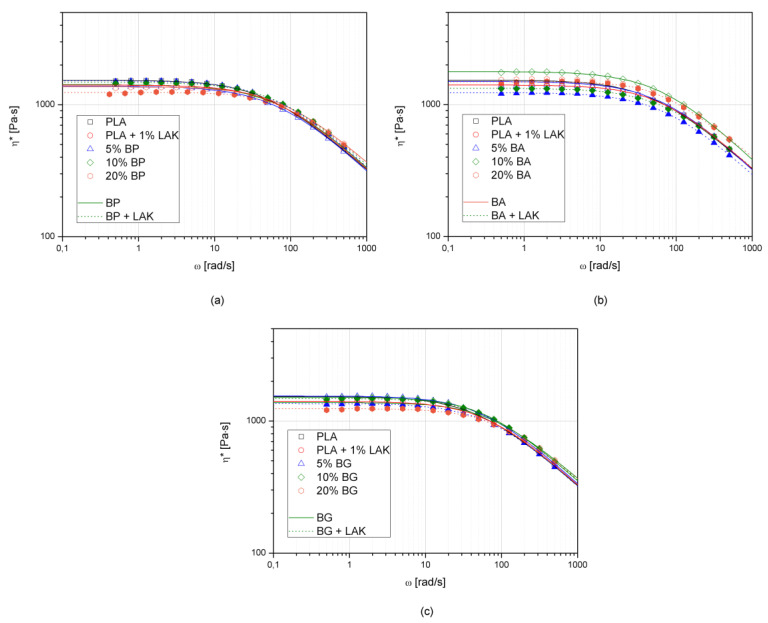
Complex viscosity curves of PLA and its composites filled with BP (**a**), BA (**b**), and BG (**c**).

Rheological properties of PLA-basalt powder composites were evaluated using oscillatory rheometry. Figure 3 shows the complex viscosity changes as a function of angular frequency. Additionally, in Table 1, the rheological parameters such as zero shear viscosity (η_0_) calculated after fitting experimental data to the Carreau–Yasuda model [61] and information about cross over point of storage (G′) and loss modulus (G″) curves are presented. The incorporation of inorganic particle-shaped fillers such as silica [62], calcium carbonate CaCO_3_ [63], or barium sulfate BaSO_4_ [64] usually causes viscosity growth. In most filled polymers, in the case of reaching the rheological percolation threshold, the Newtonian plateau disappears gradually with increasing filler content [62,65]. The reciprocal effect of lowered complex viscosity caused by the addition of organoclays to PLA was discussed by Acik et al. [66]. The authors explained the decreased viscosity observed for several grades of montmorillonite-filled composites in comparison to the unmodified polymer as a low degree of filler intercalation and limited interparticle interactions. In contrast, Liu and coworkers [67] described in their work a decrease in complex viscosity after the addition of a small amount of nanometric modified SiO_2_ due to strong interactions with polyethylene macromolecular chains. The addition of basalt powder showed no negative effect on the rheological properties of PLA. According to our previous studies [59], the effect of lowered viscosity relates to interactions between basalt particles and polypropylene polymeric chains in a molten state, which probably also occurs in the case of PLA–BP composites. A lack of shear-thinning at low angular frequencies of the filled polymeric system may be interpreted as confirmation of its good compatibility. Only the 10BA sample showed increased viscosity in the entire considered angular frequency range. Composites filled with 20 wt% of the filler from series BP and BG showed lowered viscosity in comparison to non-modified PLA and PLA–LAK. Moreover, lowered viscosities for materials nucleated with LAK suggest the plasticizing effect of the nucleating agent on PLA.

Changes of the cross-over point may not only be related to changes in molecular weight and molecular weight distribution of the tested polymer [68]. Higher values of ω at the cross-over point correspond to lower relaxation times of the polymer. Cross over points of G′ and G″ were not observed in the case of non-nucleated samples for 20BP and 20BG in the considered ω range, while for the nucleated samples, this effect was additionally noted for the 10BA and 20BA composite series. On the basis of the obtained results, it can be stated that polymeric chain movements are not limited by the presence of micrometric-sized basalt powder, and lowered complex viscosity may be a result of interactions between the basalt particles and PLA chains. It cannot be ignored that, due to low-scale hydrolytic degradation, low molecular products are present in a molten composite bulk and cause a lowered viscosity effect.

**Figure 4:**
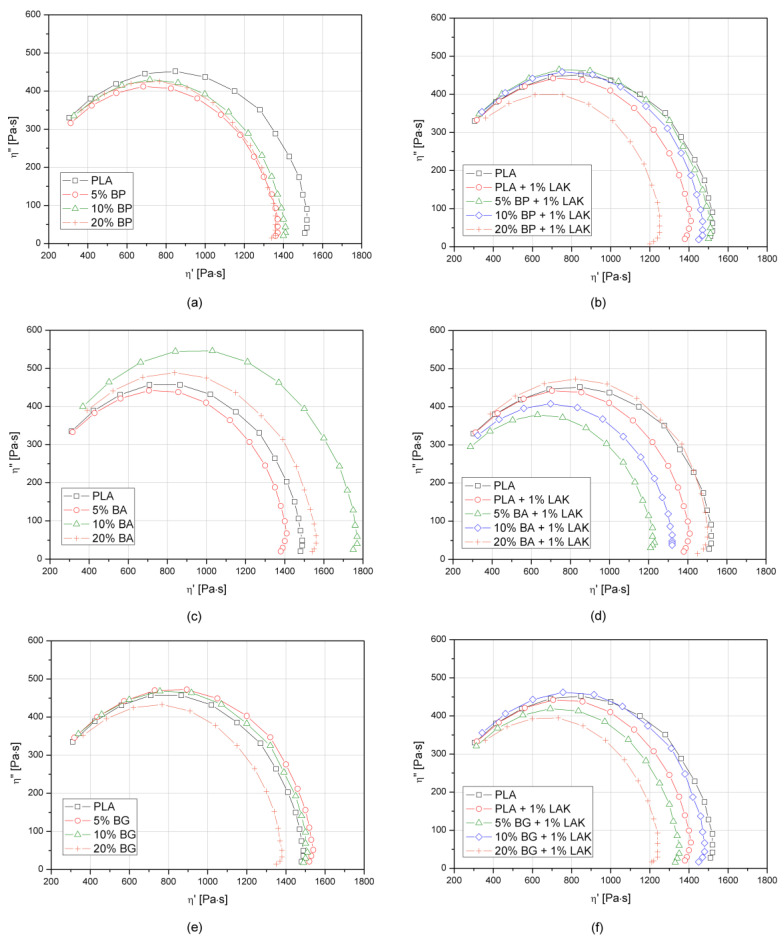
Cole–Cole plots of PLA and its composites based on the oscillatory rheological test, T = 200 °C. (**a**) PLA + **BP**; (**b**) PLA + **BP** + 1% LAK; (**c**) PLA + **BA**; (**d**) PLA + **BA** + 1% LAK; (**e**) PLA + **BG**; (**f**) PLA + **BG** + 1% LAK.

The miscibility in the molten state of PLA-based composites filled with basalt powder modified with two different organosilanes was determined by using two rheological analyses (i.e., presentation of oscillatory test results in the form of Cole–Cole and Van Gurp–Palmen plots). Both methods are useful for rheological data presentation, which describes the miscibility of polymeric blends [69,70,71,72,73] but also determines the compatibility of thermoplastic composites [73,74]. Figure 4 shows the Cole–Cole plots of the PLA–BP/BA/BG composites as changes of imaginary part viscosity (η″) as a function of the real part viscosity (η′). A smooth semicircle shape of the Cole–Cole graphs suggests good compatibility of the two-phase system in a molten state. All the considered samples showed a lack of deviation from the course of the curve obtained for neat PLA, which may be interpreted as their overall good homogeneity and compatibility. According to Hoseini et al. [72], the semicircle curves with a larger radius reflect the creation of compatible suspensions with fine morphology, which was observed for the BA and BG non-nucleated composites containing 10 and 20 wt% of the filler. For all of the composite series, the addition of LAK caused a plasticizing effect. Zhou and coworkers [75] proposed that the lowered position of the Cole–Cole plot was an effect of lubrication induced by the presence of silsesquioxanes dispersed in polypropylene. According to this finding, the observed lowering of both components in the complex viscosity of the semicircle PLA-BP/BA/BG plots concludes that LAK is not only a nucleating agent, but also a lubricating and coupling agent for the composite structures.

**Figure 5:**
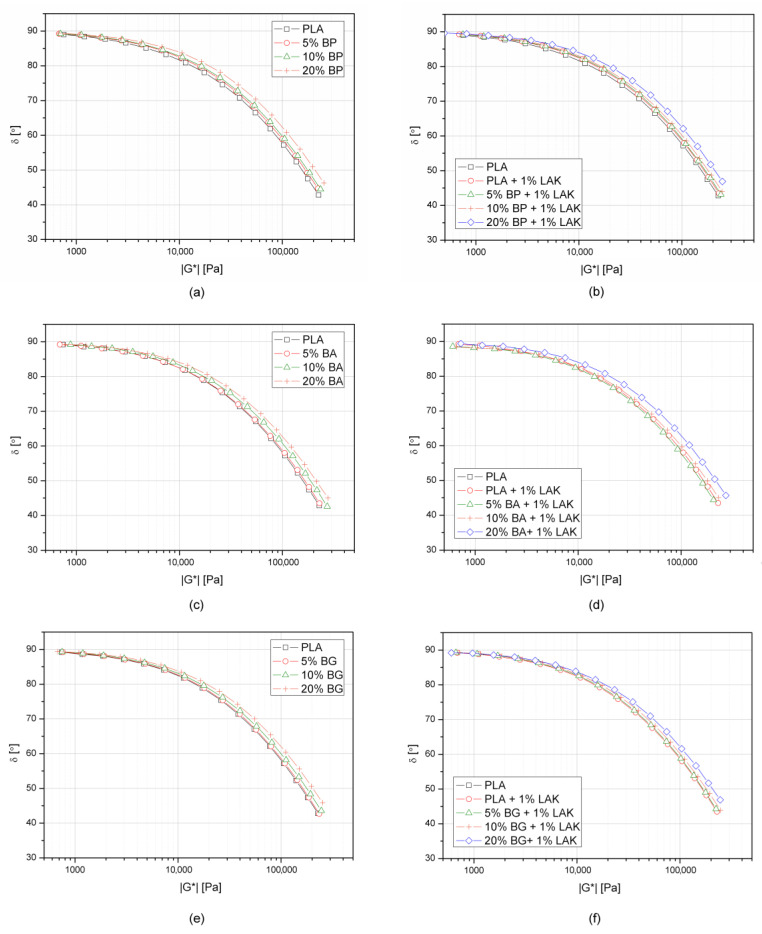
Van Gurp–Palmen plots of PLA and its composites based on the oscillatory rheological test, T = 200 °C. (**a**) PLA + **BP**; (**b**) PLA + **BP** + 1% LAK; (**c**) PLA + **BA**; (**d**) PLA + **BA** + 1% LAK; (**e**) PLA + **BG**; (**f**) PLA + **BG** + 1% LAK.

Figure 5 shows the Van Gurp–Palmen plots that present changes of phase angle (δ) as a function of complex modulus (G*). The phase angle is the phase difference between the applied strain and measured stress. In the case of a fully elastic material, the stress and strain waves are in phase (δ = 0°), whereas a strictly viscous material has two waves that are out of phase (δ = 90°) [73]. This form of presentation is more sensitive for the detection of the filler percolation threshold than the Cole–Cole analysis [76]. The creation of 3D solid-like structures of the hindered particles that are in mutual contact results in a deviation from the course of the δ(G*) curve, which for the unfilled polymer showed a continuous decrease with an increasing complex modulus value [74,76]. The rheological results presented in Figure 5 show that most of the curves achieved for neat PLA and its composites overlapped. This means that all composites in the molten state showed strong viscous-like behavior. The composites containing the highest amount of the filler (20 wt%) showed a shift in the curves to higher G* values and an even lower slope of the curve, which suggests a more viscous behavior than pure PLA. The lack of the changes in the course of the Van Gurp–Palmen plots confirmed the good compatibility of the composites, regardless of the type of surface modification, and confirmation that a network-like structure did not occur in any tested composite sample.

The presented almost complete lack of changes in the rheological and processing properties of PLA modified with basalt powder subjected to silanization using compounds with different functional groups proves the favorable processing properties of PLA. The results are in line with previous work on basalt powder-filled composites [59]. Therefore, it can be concluded that the observed test results were not accidental and constitute a special feature of basalt dust, which enables the production of composite materials without significant changes in the processing properties of the unmodified polymer.

### 3.2. Structural Analysis

**Figure 6:**
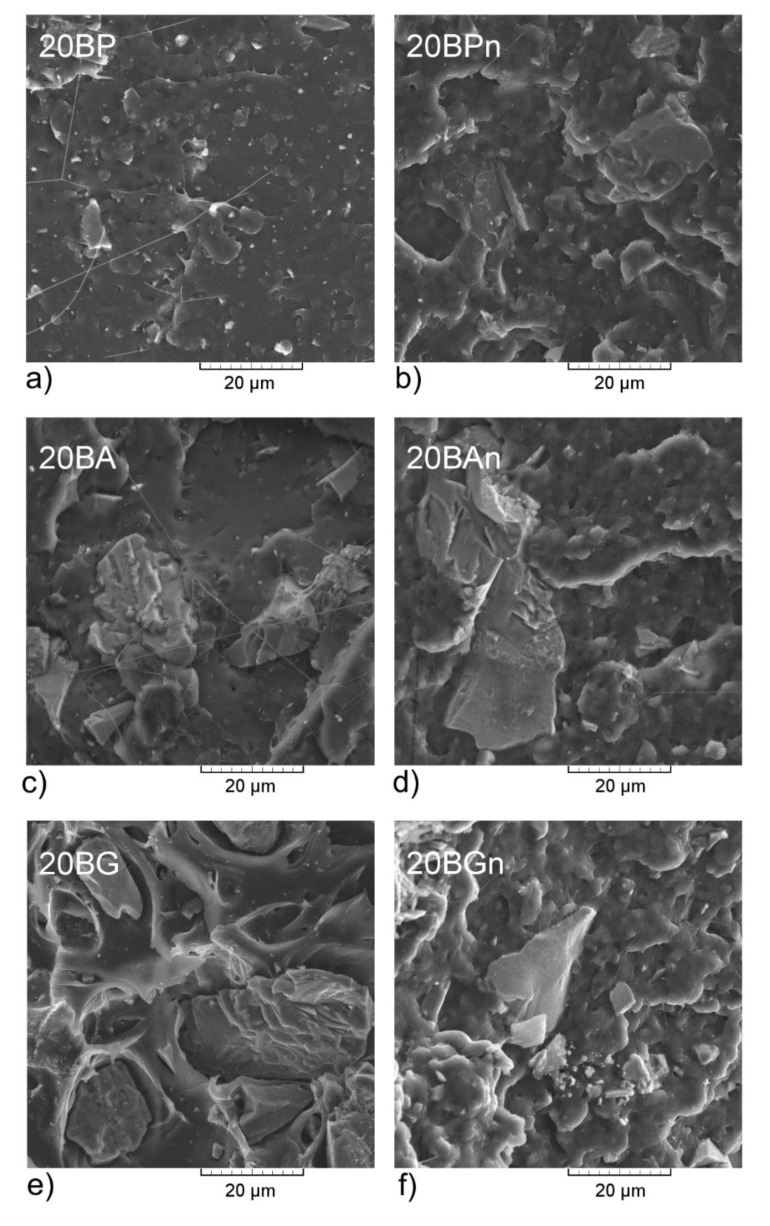
Scanning electron microscopy (SEM) images of PLA composites containing 20 wt% basalt powder. (**a**) 20BP; (**b**) 20BPn; (**c**) 20BA; (**d**) 20BAn; (**e**) 20BG; (**f**) 20BGn.

The microstructures of the brittle fracture surfaces of the chosen composites evaluated by scanning electron microscopy (SEM) are presented in Figure 6. The samples with the highest basalt powder content were chosen for the microscopic observations to check the presence of the filler aggregates. Fiber-like structures observed in the case of non-nucleated samples were fibrillated PLA, which occur during rupture breakage of the sample and heating of the amorphous polymeric matrix. In the case of the BG-filled composites, this phenomenon was not observed, which may suggest partial stabilization of the polymer by the presence of the reaction between the hydroxyl groups of PLA and epoxy groups of the GPS bonded at the surface of BG [77]. In the case of nucleated samples, a more developed surface of the broken sample was noted. This phenomenon corresponds to the plastic deformation of the PLA crystalline structures [78] and confirms that the application of LAK enhances the crystallinity of polylactide. For composites manufactured without LAK, the typical brittle fracture characteristic for amorphous thermoplastic polymers was observed [79]. The filler particles were easily visible in the examined area. For all the composites, saturation of the basalt particles was correct, which suggests good adhesion between the polymer and an inorganic filler. Based on the observations of the structure of fractures in composites, it can be concluded that the smallest number of defects, in the form of gaps in the interfacial region, occurs in the samples filled with the BA series. In the case of PB and BA, no pull-outs of the inorganic particles were observed. Moreover, it should be noticed that the silanization of the BP did not influence its tendency to agglomerate in the PLA matrix. The adhesion of the filler and the polymer seemed to be worse than in the case of the non-nucleated sample where a barely-noticeable gap at the interface could be spotted. This may have originated from the plastic deformation of the semicrystalline matrix during the sample’s fracture. Even though the affinity of basalt powder is not as low as in the case of highly hydrophilic lignocellulosic fillers [80], it is not perfect and could be enhanced. The silanization of basalt powder with 3-APS did not influence the appearance of the fracture surface of PLA. In the case of the non-nucleated sample, it was smooth, and for the LAK-modified sample, it was more irregular, indicating brittle and plastic fracture, respectively. The filler particles in both cases were embedded in the polymeric matrix and even partially covered with PLA, which indicates that treatment with 3-APS improved basalt affinity to this polymer. The microstructure of the BG-filled samples appeared different. The fracture surface of the non-nucleated composite was still smooth and in the case of the addition of LAK it was rougher, but there was also a visible gap at the filler–polymer interface that was especially prominent in the amorphous sample. It can be concluded that the treatment of basalt powder with GPS was not as effective as in the case of 3-APS, but the addition of the nucleating agent could reduce the disadvantageous influence of this silane. Based on the SEM analysis as well as the rheological assessment, it can be stated that the surface treatment of basalt particles does not cause an increased tendency to create agglomerated structures of the filler in a polymeric matrix. Neither the molten state experiments nor evaluation of the samples after solidification revealed lowered compatibility of the PLA–basalt powder composites.

### 3.3. Physical, Mechanical, and Thermomechanical Properties

The mechanical and physical properties of the polylactide-based composites filled with different grades of basalt powder and the nucleating agent are collected in Table 2. The density of the unmodified PLA was 1.23 g/cm^3^ and all composite samples showed higher *ρ* values. This result is reasonable as the density of basalt powder was about 2.91 g/cm^3^ [81]. The *ρ* values measured for the nucleated series were also higher in comparison with their counterparts without LAK. As the amorphous phase is less dense than the crystalline one, the increase in density due to the addition of this nucleating agent is presumably caused by the improved crystallinity [82]. Apart from the density of the filler and modification of the matrix, another factor that can influence the specific gravity of the composite material is the presence of gaps and porosities, especially at the interface. Therefore, if the basalt powder silanization improves the interactions between the polymer and the filler, it also should have an indirect effect on the material’s density. For the filler contents of 10 and 20 wt%, the BP and BA-filled samples showed almost the same density, whereas the ones containing BG were less dense. This outcome can be observed for both the nucleated and unmodified series. It can be concluded that the silanization of basalt powder with GPS is less effective than modification with 3-APS in terms of the reduction in gaps at the interface, which is in line with the results of the SEM observations.

Mechanical properties of the PLA-based composites also changed due to the addition of different types of basalt powder and the LAK nucleating agent. The tensile strength of the unmodified PLA was about 64.1 MPa, which is typical for this polymer [83]. The nucleated polylactide showed a lower Rm value, but the difference of 1.6 MPa was small, considering the measurement uncertainties. This result is not surprising as the study by Wang et al. showed that the influence of the nucleating agent on the tensile strength of PLA was small [84]. More distinct changes were caused by the addition of BA, BG, or BP. Incorporation of the particulate filler was characterized by low aspect ratio, resulting in a gradual decrease in tensile strength to about 55 MPa for both the amorphous and the crystalline samples. A similar relationship was described in the case of PLA filled with natural extract powders [85] and peanut shell powder [86] as well as wood, cork, and bamboo dust [87]. The decrease in Rm of a composite due to the addition of a filler is often explained by insufficient adhesion between the phases, so the silanization of the basalt powder should improve the tensile strength of the material. In fact, the BA-filled samples showed the highest Rm values among the specimens with the corresponding filler content. Apart from the low affinity of the filler and the matrix, the decrease in tensile strength can be connected to the low aspect ratio of the basalt powder, which does not allow for efficient stress transfer and therefore the filler particles can act as points of stress concentration [88]. The most notable difference can be noticed for the composites containing 20 wt% and 1% LAK, in this case, the composite with basalt powder modified with 3-APS was 3.8 MPa stronger than the one filled with BP. The samples filled with BG were characterized by a less favorable Rm value than the ones with BA; up to 10 wt% of the filler, their tensile strength was similar to the ones containing unmodified basalt powder and for the 20BG/LAK sample, it was still lower than in the case of modification with 3-APS.

Both the addition of the nucleating agent and the basalt powder caused an increase in the modulus of elasticity of the samples, but the pattern of the changes was different. Modification of PLA with the nucleating agent caused an about 20% increase of the E value, which is a common result [89] caused by a higher content of densely packed crystalline phase in the material [44]. The addition of basalt powder also increased the modulus of PLA; however, its content needed to be 20 wt% to get a similar outcome to 1 wt% LAK. The increased crystallinity of the nucleated polymer enhances intramolecular interactions [84], which effectively improves the mechanical properties of the material. Micrometric particles interact with the polymer on a different level and the increase in elasticity modulus due to its addition was mostly caused by the fact that the E value of basalt was about 89 GPa [90], which was 36 times higher than for the pure PLA. The stiffening effect of basalt powder also depended on the interactions between the filler and the polymer; their strong interactions limit the movement possibilities of the particles, thus increasing the elasticity modulus. For the non-nucleated samples, no correlation of the basalt treatment method and E value were found and, taking into consideration the measurement uncertainties, it can be concluded that there was no difference between the samples with the same content of various filler grades. In the case of the nucleated series, the composites containing up to 10% of BG and BA had a higher modulus than the ones with BP, but the samples with the highest filler content showed the opposite behavior. Nevertheless, all the differences were insignificant, taking into consideration the measurement errors. Therefore, it can be concluded that nucleation of PLA is the most efficient way to improve its tensile modulus. Addition of rigid basalt powder also can increase the E value, but the silanization of the filler does not have a visible influence on this property.

Polylactide is known for its low elongation at the break value and the ε_b_ value of 4.7% denoted for the pure PLA is a typical value [91]. Nucleation of PLA with 1 wt% LAK did not significantly change this property. Application of different kinds of basalt powder to both nucleated and unmodified polylactide caused a visible reduction in elongation at break, but the magnitude of the changes was not correlated with the filler content. For example, the ε_b_ value of the 5BP was 3.6% and one of the 20BP samples was only 0.4% lower. The decrease in elongation at break, along with the aforementioned reduction in tensile strength, is a common effect of low aspect ratio filler application [92,93]. The filler particles, which are loosely connected to the polymeric resin, can serve as points of stress concentration, and facilitate the propagation of cracks [94]. Interestingly, the elongation at break of the BA-filled non-nucleated composites was notably higher in comparison to their counterparts containing BP. This result can be caused by a better affinity between PLA and the silanized filler [92] as well as a plasticizing effect of the low molecular weight 3-APS [95]. It also needs to be noted that in the case of the nucleated samples, the difference in the ε_b_ value between the composites with different basalt grades was much smaller. It can be concluded that the modifying effect of LAK was prominent enough to cover up the influence of filler chemical treatment.

The hardness of pure PLA determined by the ball indentation method was about 140.9 MPa. As basalt is a hard rock (5–9 in the Mohs’s Scale, depending on the resource [96]), its application caused an increase in PLA hardness up to 150.5 MPa for the sample filled with 20 wt% BP. Silanization of the filler caused an even more prominent hardness growth, especially in the case of the 3-APS-modified specimens. This resulted from an enhancement of the matrix–filler interactions [97]. Similar effects were reported by Nishitani, Kajiyama, and Yamanaka in the case of polyamide composites filled with silane treated hemp fiber [98]. On the other hand, the unfilled, nucleated PLA showed higher hardness than the 20BA sample. This result can be ascribed to the increased amount of the harder crystalline phase. The addition of basalt powder to the nucleated PLA had a similar effect on composite hardness, as in the case of the non-nucleated matrix. As a result, the 20BA/LAK and 20BG/LAK were characterized by a hardness of almost 180 MPa, 40 MPa higher than pure PLA. It needs to be stressed that simultaneous nucleation and silanization did not cancel their beneficial effects on the mechanical properties of PLA.

The impact strength of all the non-nucleated samples was in the range of 7.46–9.94 kJ/m^2^. Interestingly, the unfilled PLA was characterized by a slightly lower *a* value than the basalt-filled composites. The addition of 5 wt% of the inorganic filler to polylactide caused an increase in impact strength and then its value decreased gradually along with the filler content. Similar results were described, for example, in the case of calcium sulfate-filled PLA, where an initial increase in impact strength with filler content was followed by a decrease [99]. The best results were obtained for the 5BA composition, but the differences between the three basalt grades used in this study were negligible. The basalt powder particles, regardless of the silanization process or lack thereof, were well connected to the polymeric matrix and their presence increased the amount of energy needed to break the sample. However, at higher filler content, the rigid basalt particles facilitated the crack propagation, and the impact strength decreased. The nucleated series showed a similar relationship between the composition and impact strength as the amorphous series, but the distribution of the results was wider. Supposedly, this result comes from the fact that the presence of the filler increases the impact strength of PLA-based composites and the improvement in crystallinity is connected to the decrease in the *a* value [100].

The thermal properties of the PLA and its composites determined using the DSC and TGA methods are presented in Table 3. To connect the information about the material crystalline structure and its properties, the values of crystallinity determined in the first heating were analyzed. As all the samples were manufactured in the same conditions, it was assumed that their thermal history did not cause any deviation of the results.

PLA and its non-nucleated composites were characterized by X_CR_ values in the range of 30.0–53.1%. This value is quite high for polylactide unmodified with nucleating agents, but the PLA grade used in this study contained a small amount of d-isomer (<0.5%), which made it more susceptible to melt crystallization [101]. Moreover, as was shown in our previous research, untreated basalt powder, which is rich in SiO_2_, also promotes the crystallization of PLA [53]. Similar results are reported in Table 3. The addition of 5 wt% of BP to PLA caused an increase in crystallinity from 30.0% to 39.6%, and along with increasing filler content, the X_CR_ grew to 46.0%. The crystallinity of the composites containing BA was still higher than the one of the neat resin, but all the obtained values were similar and did not exceed 43.1%. The highest X_CR_ values were measured for the samples with basalt powder modified using GPS. Furthermore, all the non-nucleated materials showed cold crystallization behavior, which is typical for PLA characterized by a slow melt crystallization rate [102]. The addition of the LAK successfully reduced the cold crystallization as the polymer created the crystalline structure during cooling from the melt. All the nucleated composites were characterized by higher X_CR_ values than their LAK-free counterparts. In this case, the crystallinity degree was independent of the basalt powder grade or content. Therefore, it was concluded that the modification of PLA with the LAK nucleating agent was an efficient way to enhance the crystallization rate and improve the crystallinity of both the unfilled polymer and its composites with different basalt grades. Importantly, the chemical treatment of basalt powder did not affect the nucleation process in any way, so the same procedure can be applied to increase the crystallinity of various composites.

The values of 5% mass loss temperature (T_5_) and degradation temperature (T_DEG_) determined using the TGA method are also shown in Table 3. Unfilled PLA had T_5_ and T_DEG_ values of 329.30 °C and 359.20 °C, respectively. The addition of basalt powder, regardless of its kind, resulted in a slight decrease in both temperature values; however, thermal degradation of PLA took place above 300 °C, which is at least 120 °C higher than the melting temperature. It can be assumed that the addition of either kind of basalt powder did not reduce the processing window of this polymer. In the case of the nucleated series, the results were even more advantageous. All the tested samples had T_5_ values of about 324–328 °C and T_DEG_ values of 357–361 °C. Presumably, as the crystallinity of the nucleated composites was also relatively uniform, it was the main factor influencing their thermal degradation.

**Figure 7:**
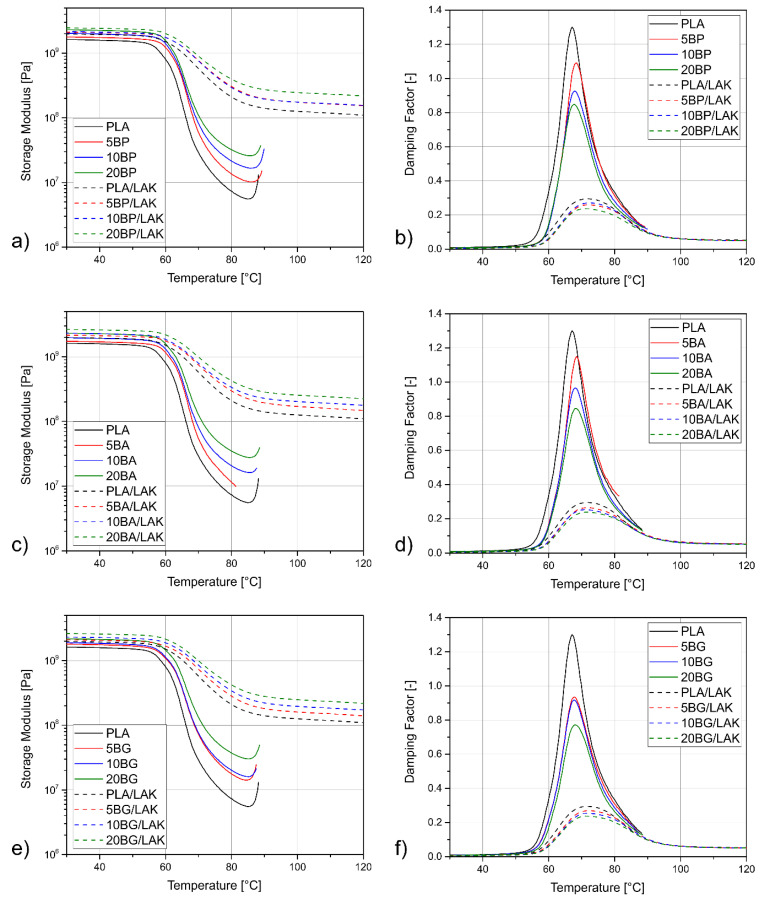
The storage modulus (**a**,**c**,**e**) and damping factor (**b**,**d**,**f**) curves of PLA and its composites.

The storage modulus (G′) and damping factor (tanδ) curves as a function of temperature (T) determined for PLA and its composites with basalt powder and/or nucleating agent are shown in Figure 7. For all the non-nucleated materials, the run of the G′ vs. T was similar. At the beginning, the values of storage modulus were high (above 109 Pa), as the material was in a glassy state. Above 50–60 °C, a sudden drop of G′ was attributed to the relaxation of the amorphous part of the polymer [44]. This phenomenon was also indicated by a prominent peak in the tanδ vs. T curve. A rubbery plateau was not developed, as around 90 °C, the storage modulus increased once again, indicating cold crystallization of the polymer [103]. Even though all the PLA-based materials underwent similar processes during the experiment, their thermomechanical properties were not the same. Unfilled PLA had the lowest storage modulus among all the studied samples. This observation agrees with the results of the quasi-static tensile test, which proved that the addition of basalt powder increased the stiffness of polylactide. Furthermore, despite the comparable glass transition temperatures for the PLA and PLA-BP non-nucleated composites and an increase of only about 1 °C after BP addition, the run of the G′ curve indicated that the basalt-filled samples had higher stiffness after the glassy transition due to the reinforcing effect of the rigid domains of the filler dispersed in the polymeric matrix. This phenomenon indicates a beneficial influence of this filler on the thermomechanical stability of the composites even in the case of non-simultaneous use of the nucleating agent.

Even though the storage modulus of the nucleated PLA and its composites determined at lower temperatures was similar in the unmodified series, its further run was different. The drop of G′, attributed to the glass transition of the material, took place at higher temperatures. Moreover, the LAK-nucleated samples did not undergo cold crystallization and after the relaxation of the amorphous phase, the G′ value remained stable up to the end of the measurement. The storage modulus of the crystalline samples measured above the glass transition was about one order of magnitude higher than the non-nucleated ones. This behavior results from the fact that the LAK-modified samples contained a lower amount of the amorphous phase that underwent relaxation, and also from enhanced interactions of the polymeric chains in the amorphous and crystalline parts. Interestingly, the nucleated samples filled with different kinds of basalt showed similar behavior during the DMTA experiment, but the G′ vs. T curves of the samples with higher basalt content were shifted vertically to higher storage modulus values. This result indicates that the addition of this inorganic particle-like filler improved the thermomechanical stability of the crystalline samples as well as the non-nucleated ones.

In order to quantitatively evaluate the influence of the filler type and content on the thermomechanical properties of the PLA-based composites, the values of glass transition, tanδ_max_, brittleness B, the effectiveness of the filler C, and reinforcement efficiency r are presented in Table 4. Glass transition of the unmodified PLA was about 67.1 °C. The addition of the basalt powder, regardless of its kind, shifted it to slightly higher temperatures. Even though this change was rather small, it indicates that there are interactions of the filler and the matrix and that the basalt particles limit the movement possibilities of the polymer chains. This result was also true for the silanized filler. The application of the modifying agent characterized by low molecular weight did not cause plasticization of the polymer. The nucleated materials underwent a glass transition at a higher temperature than the LAK-free ones, which was caused by their higher crystallinity. The T_G_ value determined for the PLA and its composites was almost the same, about 71.0 °C, and the deviations were probably caused by random factors such as signal processing by the measuring system. It can be concluded that the crystallinity had a bigger influence on the glass transition of PLA-based composites than the addition of the filler. Therefore, the interactions of the macromolecules in the amorphous and the crystalline phase of the material were stronger than the interactions of the polymeric chains and the filler. The highest value of tanδ_max_ (that is, the damping factor value at T_G_) was determined for the unmodified, unfilled PLA. The addition of basalt powder caused a decrease of the tanδ_max_ value and the magnitude of the change was directly proportional to the filler content. This relationship was also true for the nucleated series, but in this case, the peak of the damping factor was much lower. No visible difference in the samples with the untreated and silanized basalt powder was noticed.

The C parameter, called the effectiveness of the filler, helps to evaluate the influence of the filler on the thermomechanical stability of the composite. The lower its value, the more effective the filler. As shown in Table 4, the C values decreased along with the filler content, especially in the case of the non-nucleated samples filled with the silanized basalt powder. The lowest value of 0.26 was calculated for the 20BG sample. It was visibly smaller than in the corresponding sample with untreated basalt, which indicated that silanization with GPS had a positive effect on the thermomechanical properties of the PLA composites. Nevertheless, the nucleated series presented notably higher C values in the range of 0.66–0.79. In this case, the effectiveness of the silanized filler was not better than the untreated basalt. However, this result did not indicate poor thermomechanical stability of the studied materials, but showed that the application of the filler had a smaller influence on this property than the matrix.

Unlike the C factor, the value of the reinforcement efficiency r was related to the volumetric filler content. For the BP-filled non-nucleated series, it was seen that the composite filled with 10 wt% of basalt powder showed the highest r values at 30 °C and 80 °C (r_30°C_ and r_80°C_), whereas the 5BP and 20BP samples showed comparable reinforcement efficiency. A similar relationship was observed for the composites filled with BA, but in this case, the r values calculated for the samples with 10 and 20 wt% of the filler were much more similar. It can be assumed that modification of basalt powder with 3-APS allowed application of a higher content of this low-cost waste filler in the PLA composites without deterioration of its properties. In the case of the GPS-modified basalt powder, the samples with 10 and 20 wt% of the filler had a similar reinforcement efficiency as the BA-filled composites, but the highest r-value was achieved by the 5BG sample. This result indicated that even a small amount of this filler had a noticeable influence on the thermomechanical properties of the PLA-based composites, so the chemical treatment of basalt powder with GPS was a reasonable solution. Furthermore, in the case of all non-nucleated samples, the r values calculated at 80 °C were an order of magnitude higher than the ones evaluated at 30 °C. It can be concluded that the application of basalt powder, regardless of its chemical modification, can significantly improve the high-temperature performance of polylactide-based composites. The reinforcement efficiency of the nucleated samples determined at 30 °C was lower than for those unmodified with LAK. Even though the change was not very prominent, it showed that the influence of nucleation on the PLA thermomechanical properties was stronger than the influence of the addition of the filler. For the r_80°C_ value, the difference between the amorphous and crystalline samples was even more noticeable as the increase in crystallinity was the most efficient way to improve the high-temperature stability of PLA.

Brittleness, as proposed by Brostow et al., is a property of a material that combines its quasi-static and dynamic performances. The lower the brittleness, the better the ductility of the specimen [104]. The addition of the unmodified basalt powder increased the B value of PLA. This result is reasonable if the decrease in tensile strength and elongation at break of the BP-filled composite is taken into consideration. Interestingly, the 3-APS-modified series showed much lower brittleness, and the B value determined for the 20BA sample was even lower than the one of the unfilled PLA. This outcome indicates enhanced adhesion between the phases in the composite, as the material can present more ductile behavior without cracking. The BG-filled series was less brittle than the composites with the unmodified basalt powder, but not as ductile as the samples containing BA. Therefore, the modification of the filler with GPS was only partially effective. The nucleated composites presented higher B values than the amorphous ones. Interestingly, both series containing the signalized basalt powder showed similar brittleness, which was lower than for BP-filled composites, but higher than for the PLA/LAK sample.

**Figure 8:**
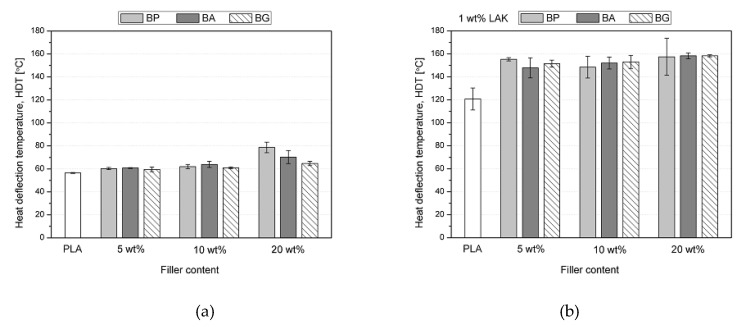
Heat deflection temperature: (**a**) of the non-nucleated PLA composite samples; (**b**) of the nucleated PLA composite samples.

The value of the heat deflection temperature (HDT) was used to evaluate the thermomechanical stability and to determine the temperature range for PLA applications. In Figure 8, the HDT measured for the unmodified and nucleated PLA composites with basalt powder is shown. The HDT of unfilled, non-nucleated polylactide did not exceed 60 °C, which is typical for this material and can be connected to the glass transition temperature of about 67 °C, as indicated by the DMTA. The addition of 5 and 10 wt% basalt powder did not visibly change the heat deflection temperature of the composites. Only the samples with the highest studied filler content were characterized by HDT above 70 °C. Interestingly, this behavior was the most visible for the BP series. Figure 8 indicates that nucleation with LAK was a much more efficient way to improve the heat deflection temperature of PLA than the addition of the chemically treated basalt powder. The unfilled, nucleated PLA had an HDT value about twice as high as the amorphous sample. This result agrees with the literature, which shows that the increase in crystallinity resulted in improved thermomechanical stability of the polylactide [44]. However, the highest heat deflection values up to 160 °C were found in the composites containing both LAK and basalt powder. As no relationship of the filler content and the crystallinity of the basalt-filled nucleated samples was identified, this increase should be attributed to the presence of the filler particles, which are characterized by high thermal stability and good affinity to the polymeric matrix. By limiting the movement of PLA chains, the basalt particles improved the high-temperature stability of the composites. In the case of the non-nucleated series, this behavior was also true, but the interactions of the filler and the polymer were not strong enough to suppress the relaxation of the amorphous phase, which determines the HDT value. For the nucleated, crystalline composites, the glass transition was not the main phenomenon behind their thermal stability and the filler–polymer interactions added an extra boost to the high-temperature behavior of the composites. Interestingly, there was no clear relationship between the filler content and the HDT value of the PLA-based composites. Even 5 wt% of the filler had an observable effect on the thermomechanical stability of the samples, but its increase to 20 wt% did not have a disadvantageous influence. Silanization with either 3-APS and GPS also did not affect the heat deflection temperature of PLA.

## 4. Conclusions

In this study, PLA-based composites filled with different grades of basal powder as well as a nucleating agent were successfully manufactured and subjected to an in-depth analysis of structure, mechanical properties, miscibility, and thermomechanical stability. It was found that adding up to 20 wt% of the inorganic waste filler as well as a nucleating agent resulted in excellent thermomechanical stability (HDT up to 160 °C) and mechanical properties (tensile modulus about 3 GPa, impact strength about 2.5 kJ/m^2^) and does not limit processability of the material. Silanization with either 3-APS or GPS can, to some extent, improve the matrix–filler interactions in the composite, regardless of the application of the nucleating agent. Even though the silanization procedure was performed correctly, the improvement in the properties of the composites due to chemical treatment with basalt powder does not justify the use of the silanization step in the preparation procedure. Apparently, basalt powder, due to relatively low number of free –OH groups on the surface, is not as prone to improvement of its affinity to PLA as some of the more hydrophilic materials such as plant fibers. BP, without time-consuming chemical treatment, can be an effective filler for thermally-stable, easy to process, sustainable PLA composites.

## Figures and Tables

**Table 1:** Rheological parameters of PLA–BP/BA/BG.

Material	Non-Nucleated			Nucleated		
	η_0_	G′ = G″	ω at G′ = G″	η_0_	G′ = G″	ω at G′ = G″
	[Pa·s]	[Pa]	[rad/s]	[Pa·s]	[Pa]	[rad/s]
PLA	1535	142,100	404	1409	150,400	432
5BP	1385	154,400	484	1521	153,800	418
10BP	1421	161,600	476	1479	165,200	454
20BP	1368	-	-	1240	-	-
5BA	1503	144,300	401	1233	141,700	473
10BA	1783	170,500	395	1332	-	-
20BA	1564	194,900	500	1494	-	-
5BG	1546	148,000	398	1356	153,200	468
10BG	1513	161,800	437	1480	165,400	451
20BG	1381	-	-	1238	-	-

**Table 2:** Mechanical and physical properties of the PLA-BP/BG/BA composites.

Material	Tensile Strength, Rm	Elasticity Modulus, E	Elongation at Break, ε_b_	Impact Strength, *a*	Hardness	Density, *ρ*
	[MPa]	[GPa]	[%]	[kJ/m^2^]	[MPa]	[g/cm^3^]
PLA	64.1 ± 1.3	2.48 ± 0.05	4.7 ± 0.1	7.46 ± 0.69	140.9 ± 3.3	1.23 ± 0.009
5BP	59.4 ± 0.8	2.52 ± 0.05	3.6 ± 0.1	9.59 ± 1.04	142.0 ± 4.5	1.29 ± 0.003
10BP	57.3 ± 0.9	2.71 ±0.05	3.3 ± 0.2	8.89 ± 1.69	144.5 ± 2.6	1.31 ± 0.009
20BP	54.8 ± 0.6	2.94 ± 0.10	3.2 ± 0.2	7.54 ± 0.69	150.5 ± 4.7	1.39 ± 0.012
5BA	60 ± 1.4	2.59 ± 0.16	3.9 ± 0.1	9.23 ± 0.79	144.3 ± 3.2	1.27 ± 0.014
10BA	58.6 ± 1.1	2.75 ± 0.06	3.7 ± 0.3	8.78 ± 1.08	145.8 ± 3.6	1.31 ± 0.008
20BA	56.6 ± 0.3	2.80 ± 0.15	3.8 ± 0.5	7.59 ± 0.52	157.1 ± 4.5	1.39 ± 0.008
5BG	58.0 ± 0.9	2.63 ± 0.06	3.7 ± 0.2	9.94 ± 1.49	142.4 ± 7.5	1.28 ± 0.010
10BG	58.0 ± 0.9	2.66 ± 0.10	4.0 ± 0.3	8.46 ± 0.68	142.8 ± 7.9	1.31 ± 0.005
20BG	55.5 ± 0.6	3.07 ± 0.06	3.1 ± 0.1	7.79 ± 0.62	154.7 ± 4.5	1.38 ± 0.011
PLA/LAK	62.5 ± 0.3	2.95 ± 0.05	5.0 ± 0.6	7.46 ± 1.41	157.7 ± 7.4	1.24 ± 0.009
5BP/LAK	59.1 ± 0.9	3.04 ± 0.02	2.9 ± 0.2	9.54 ± 1.51	163.2 ± 7.6	1.28 ± 0.007
10BP/LAK	56.7 ± 0.4	3.14 ± 0.04	3.1 ± 0.3	8.41 ± 1.31	164.8 ± 5.3	1.32 ± 0.005
20BP/LAK	52.3 ± 0.5	3.25 ± 0.05	2.6 ± 0.1	6.91 ± 0.71	174.2 ± 5.1	1.41 ± 0.007
5BA/LAK	59.7 ± 1.0	2.95 ± 0.18	3.3 ± 0.3	9.8 ± 1.52	163.6 ± 6.0	1.29 ± 0.004
10BA/LAK	58.2 ± 0.6	3.1 ± 0.08	3.1 ± 0.3	9.39 ± 2.6	172.1 ± 4.2	1.32 ± 0.006
20BA/LAK	56.1 ± 0.6	3.38 ± 0.08	2.9 ± 0.3	9.09 ± 4.48	177.8 ± 4.7	1.41 ± 0.006
5BG/LAK	59.0 ± 0.9	2.91 ± 0.15	3.4 ± 0.3	11.53 ± 4.76	167.4 ± 5.5	1.29 ± 0.009
10BG/LAK	56.9 ± 1.7	3.04 ± 0.04	3.3 ± 0.2	8.56 ± 0.82	173.4 ± 3.6	1.31 ± 0.007
20BG/LAK	55.7 ± 0.7	3.38 ± 0.09	2.8 ± 0.2	6.65 ± 0.59	177.5 ± 4.9	1.40 ± 0.008

**Table 3:** Thermal properties of the PLA-BP/BG/BA composites.

Material	T_CC_	T_CR_	T_M_	X_CR_	T_5_	T_DEG_
	[°C]	[°C]	[°C]	[%]	[°C]	[°C]
PLA	101.80	96.40	180.40	30.0	329.30	359.20
5BP	99.10	95.80	177.20	39.6	325.50	356.00
10BP	100.10	96.70	177.30	45.4	323.90	353.70
20BP	100.59	97.90	177.30	46.0	316.10	349.50
5BA	96.10	95.70	177.50	38.8	325.00	357.80
10BA	97.80	94.30	177.80	43.1	322.70	353.10
20BA	97.50	97.60	176.90	42.0	319.70	346.30
5BG	97.00	95.10	178.30	37.8	324.50	357.20
10BG	97.40	97.40	177.40	43.5	323.60	354.50
20BG	95.00	98.60	178.00	53.1	323.30	349.40
PLA/LAK	-	130.70	181.80	45.4	326.90	361.30
5BP/LAK	-	124.70	176.20	45.1	327.30	360.70
10BP/LAK	-	126.20	176.90	44.1	326.50	358.10
20BP/LAK	-	123.40	175.60	48.9	327.00	359.8
5BA/LAK	-	125.30	176.80	45.6	324.40	357.20
10BA/LAK	-	125.70	176.80	42.0	326.30	358.90
20BA/LAK	-	123.80	175.90	49.2	325.90	359.00
5BG/LAK	-	124.50	177.20	43.9	325.30	358.10
10BG/LAK	-	124.80	176.20	45.0	326.20	360.50
20BG/LAK	-	124.40	175.60	46.4	327.70	360.10

**Table 4:** Thermomechanical properties of the PLA-BP/BG/BA composites.

Material	Glass Transition, T_G_	tanδ_max_	Brittleness B	C	r_30°C_	r_80°C_
	[°C]	[-]	[10^10^/%Pa]	[-]		
PLA	67.1	1.300	1.31	-	-	-
5BP	68.2	1.090	1.56	0.59	4.50	39.18
10BP	67.8	0.926	1.49	0.43	5.60	42.78
20BP	68.7	0.848	1.36	0.32	4.36	35.40
5BA	68.5	1.150	1.47	0.67	3.10	26.62
10BA	68.1	0.966	1.36	0.43	4.78	40.65
20BA	68.1	0.846	1.13	0.30	4.49	39.26
5BG	67.8	0.935	1.50	0.46	5.07	64.93
10BG	67.8	0.918	1.31	0.42	3.82	39.12
20BG	68.1	0.772	1.49	0.26	3.53	42.84
PLA/LAK	71.2	0.295	1.01	-	-	-
5BP/LAK	71.5	0.260	1.63	0.73	2.99	20.72
10BP/LAK	71.9	0.271	1.50	0.77	1.90	8.89
20BP/LAK	70.5	0.235	1.56	0.66	2.52	9.24
5BA/LAK	71.5	0.265	1.41	0.77	4.14	18.76
10BA/LAK	70.9	0.253	1.40	0.73	3.69	13.34
20BA/LAK	70.9	0.238	1.30	0.66	3.51	10.73
5BG/LAK	71.2	0.269	1.40	0.79	2.76	15.92
10BG/LAK	71.9	0.255	1.31	0.72	3.69	13.97
20BG/LAK	71.2	0.238	1.35	0.66	3.41	10.54

## References

[1] Moraviec B. (2018). Plastics in the circular economy (CE). Environ. Prot. Nat. Resour. Ochr./Środowiska Zasobów Nat..

[2] Czarnecka-Komorowska D., Wiszumirska K. (2020). Sustainability design of plastic packaging for the Circular Economy. Polimery.

[3] Zink T., Geyer R. (2019). Material Recycling and the Myth of Landfill Diversion. J. Ind. Ecol..

[4] Jin F.-L., Hu R.-R., Park S.-J. (2019). Improvement of thermal behaviors of biodegradable poly(lactic acid) polymer: A review. Compos. Part B Eng..

[5] Madhavan Nampoothiri K., Nair N.R., John R.P. (2010). An overview of the recent developments in polylactide (PLA) research. Bioresour. Technol..

[6] Vink E.T.H., Rábago K.R., Glassner D.A., Gruber P.R. (2003). Applications of life cycle assessment to NatureWorksTM polylactide (PLA) production. Polym. Degrad. Stab..

[7] Zhang Q., Cai H., Ren X., Kong L., Liu J., Jiang X. (2017). The Dynamic Mechanical Analysis of Highly Filled Rice Husk Biochar/High-Density Polyethylene Composites. Polymers.

[8] Członka S., Strąkowska A., Kairytė A. (2020). Application of Walnut Shells-Derived Biopolyol in the Synthesis of Rigid Polyurethane Foams. Materials.

[9] Zedler Ł., Colom X., Saeb M.R., Formela K. (2018). Preparation and characterization of natural rubber composites highly filled with brewers’ spent grain/ground tire rubber hybrid reinforcement. Compos. Part B Eng..

[10] Hao X., Yi X., Sun L., Tu D., Wang Q., Ou R. (2019). Mechanical properties, creep resistance, and dimensional stability of core/shell structured wood flour/polyethylene composites with highly filled core layer. Constr. Build. Mater..

[11] Salasinska K., Ryszkowska J. (2015). The effect of filler chemical constitution and morphological properties on the mechanical properties of natural fiber composites. Compos. Interfaces.

[12] Goudar S., Jain R.K., Das D. (2020). Physico-mechanical properties of tamarind pod shell-based composite. Polym. Compos..

[13] Leszczyńska M., Ryszkowska J., Szczepkowski L. (2020). Rigid polyurethane foam composites with nut shells. Polimery.

[14] Hejna A., Piszcz-Karaś K., Filipowicz N., Cieśliński H., Namieśnik J., Marć M., Klein M., Formela K. (2018). Structure and performance properties of environmentally-friendly biocomposites based on poly(ɛ-caprolactone) modified with copper slag and shale drill cuttings wastes. Sci. Total Environ..

[15] Fiore V., Di Bella G., Scalici T., Valenza A. (2018). Effect of plasma treatment on mechanical and thermal properties of marble powder/epoxy composites. Polym. Compos..

[16] Sahu R., Gupta M.K., Chaturvedi R., Tripaliya S.S., Pappu A. (2020). Moisture resistant stones waste based polymer composites with enhanced dielectric constant and flexural strength. Compos. Part B Eng..

[17] Gryczak M., Wong J.W., Thiemann C., Ferrari B.J.D., Werner I., Petzhold C.L. (2020). Recycled low-density polyethylene composite to mitigate the environmental impacts generated from coal mining waste in Brazil. J. Environ. Manage..

[18] Bula K., Jesionowski T. (2010). Effect of Polyethylene Functionalization on Mechanical Properties and Morphology of PE/SiO_2_ Composites. Compos. Interfaces.

[19] Mohd H.A., Abu Bakar M.B., Masri M.N., Sulaiman M.A., Amini M.H.M., Mamat S., Mohamed M. (2020). Mechanical and Thermal Properties of Hybrid Non-Woven Kenaf Fibre Mat-Graphene Nanoplatelets reinforced Polypropylene Composites. Mater. Sci. Forum.

[20] Keener T., Stuart R., Brown T. (2004). Maleated coupling agents for natural fibre composites. Compos. Part A Appl. Sci. Manuf..

[21] Hejna A., Marć M., Skórczewska K., Szulc J., Korol J., Formela K. (2020). Insights into modification of lignocellulosic fillers with isophorone diisocyanate: Structure, thermal stability and volatile organic compounds emission assessment. Eur. J. Wood Wood Prod..

[22] Hejna A., Przybysz-Romatowska M., Kosmela P., Zedler Ł., Korol J., Formela K. (2020). Recent advances in compatibilization strategies of wood-polymer composites by isocyanates. Wood Sci. Technol..

[23] Sandomierski M., Poniedziałek K., Bielicka-Daszkiewicz K., Voelkel A. (2020). Influence of diazonium and surfactant modification of the mesoporous material on its adsorption properties. Chem. Pap..

[24] Sandomierski M., Voelkel A. (2020). Diazonium Modification of Inorganic and Organic Fillers for the Design of Robust Composites: A Review. J. Inorg. Organomet. Polym. Mater..

[25] Rong M.Z., Zhang M.Q., Shi G., Ji Q.L., Wetzel B., Friedrich K. (2003). Graft polymerization onto inorganic nanoparticles and its effect on tribological performance improvement of polymer composites. Tribol. Int..

[26] Lazzara G., Cavallaro G., Panchal A., Fakhrullin R., Stavitskaya A., Vinokurov V., Lvov Y. (2018). An assembly of organic-inorganic composites using halloysite clay nanotubes. Curr. Opin. Colloid Interface Sci..

[27] Członka S., Strąkowska A., Strzelec K., Kairytė A., Vaitkus S. (2019). Composites of rigid polyurethane foams and silica powder filler enhanced with ionic liquid. Polym. Test..

[28] Shokoohi S., Arefazar A., Khosrokhavar R. (2008). Silane Coupling Agents in Polymer-based Reinforced Composites: A Review. J. Reinf. Plast. Compos..

[29] Bula K., Jesionowski T., Krysztafkiewicz A., Janik J. (2007). The effect of filler surface modification and processing conditions on distribution behaviour of silica nanofillers in polyesters. Colloid Polym. Sci..

[30] Członka S., Strąkowska A. (2020). Rigid Polyurethane Foams Based on Bio-Polyol and Additionally Reinforced with Silanized and Acetylated Walnut Shells for the Synthesis of Environmentally Friendly Insulating Materials. Materials.

[31] Członka S., Strąkowska A., Pospiech P., Strzelec K. (2020). Effects of Chemically Treated Eucalyptus Fibers on Mechanical, Thermal and Insulating Properties of Polyurethane Composite Foams. Materials.

[32] Barczewski M., Matykiewicz D., Piasecki A., Szostak M. (2018). Polyethylene green composites modified with post agricultural waste filler: Thermo-mechanical and damping properties. Compos. Interfaces.

[33] Dike A.S. (2020). Improvement of mechanical and physical performance of poly (lactic acid) biocomposites by application of surface silanization for huntite–hydromagnesite mineral. J. Thermoplast. Compos. Mater..

[34] Hatipoglu A., Dike A.S. (2020). Effects of concentration and surface silanization of barite on the mechanical and physical properties of poly(lactic acid)/barite composites. Polym. Polym. Compos..

[35] Elkawash H., Tirkes S., Hacioglu F., Tayfun U. (2020). Physical and mechanical performance of bentonite and barite loaded low density polyethylene composites: Influence of surface silanization of minerals. J. Compos. Mater..

[36] Howarter J.A., Youngblood J.P. (2006). Optimization of Silica Silanization by 3-Aminopropyltriethoxysilane. Langmuir.

[37] González-Benito J., Baselga J., Aznar A. (1999). Microstructural and wettability study of surface pretreated glass fibres. J. Mater. Process. Technol..

[38] Sever K., Sarikanat M., Seki Y., Cecen V., Tavman I.H. (2008). Effects of fiber surface treatments on mechanical properties of epoxy composites reinforced with glass fabric. J. Mater. Sci..

[39] Spencer M.W., Hunter D.L., Knesek B.W., Paul D.R. (2011). Morphology and properties of polypropylene nanocomposites based on a silanized organoclay. Polymer.

[40] Kurniawan D., Kim B.S., Lee H.Y., Lim J.Y. (2013). Effect of Silane Treatment on Mechanical Properties of Basalt Fiber/Polylactic Acid Ecofriendly Composites. Polym. Plast. Technol. Eng..

[41] Kurniawan D., Kim B.S., Lee H.Y., Lim J.Y. (2015). Towards improving mechanical properties of basalt fiber/polylactic acid composites by fiber surface treatments. Compos. Interfaces.

[42] Deak T., Czigany T., Tamas P., Nemeth C. (2010). Enhancement of interfacial properties of basalt fiber reinforced nylon 6 matrix composites with silane coupling agents. Express Polym. Lett..

[43] Ying Z., Wu D., Zhang M., Qiu Y. (2017). Polylactide/basalt fiber composites with tailorable mechanical properties: Effect of surface treatment of fibers and annealing. Compos. Struct..

[44] Nagarajan V., Zhang K., Misra M., Mohanty A.K. (2015). Overcoming the Fundamental Challenges in Improving the Impact Strength and Crystallinity of PLA Biocomposites: Influence of Nucleating Agent and Mold Temperature. ACS Appl. Mater. Interfaces.

[45] Shi X., Zhang G., Phuong T., Lazzeri A. (2015). Synergistic Effects of Nucleating Agents and Plasticizers on the Crystallization Behavior of Poly(lactic acid). Molecules.

[46] Fehri M.K., Mugoni C., Cinelli P., Anguillesi I., Coltelli M.B., Fiori S., Montorsi M., Lazzeri A. (2016). Composition dependence of the synergistic effect of nucleating agent and plasticizer in poly(lactic acid): A Mixture Design study. Express Polym. Lett..

[47] Wang Y., He D., Wang X., Cao W., Li Q., Shen C. (2013). Crystallization of poly(lactic acid) enhanced by phthalhydrazide as nucleating agent. Polym. Bull..

[48] He D., Wang Y., Shao C., Zheng G., Li Q., Shen C. (2013). Effect of phthalimide as an efficient nucleating agent on the crystallization kinetics of poly(lactic acid). Polym. Test..

[49] Qiu Z., Li Z. (2011). Effect of Orotic Acid on the Crystallization Kinetics and Morphology of Biodegradable Poly(*l*-lactide) as an Efficient Nucleating Agent. Ind. Eng. Chem. Res..

[50] Jiang L., Shen T., Xu P., Zhao X., Li X., Dong W., Ma P., Chen M. (2016). Crystallization modification of poly(lactide) by using nucleating agents and stereocomplexation. e-Polymers.

[51] Ji N., Hu G., Li J., Ren J. (2019). Influence of poly(lactide) stereocomplexes as nucleating agents on the crystallization behavior of poly(lactide)s. RSC Adv..

[52] Barczewski M., Mysiukiewicz O., Matykiewicz D., Kloziński A., Andrzejewski J., Piasecki A. (2020). Synergistic effect of different basalt fillers and annealing on the structure and properties of polylactide composites. Polym. Test..

[53] Barczewski M., Mysiukiewicz O., Matykiewicz D., Skórczewska K., Lewandowski K., Andrzejewski J., Piasecki A. (2020). Development of polylactide composites with improved thermomechanical properties by simultaneous use of basalt powder and a nucleating agent. Polym. Compos..

[54] Goodrich J.E., Porter R.S. (1967). A rheological interpretation of torque-rheometer data. Polym. Eng. Sci..

[55] Fischer E.W., Sterzel H.J., Wegner G. (1973). Investigation of the structure of solution grown crystals of lactide copolymers by means of chemical reactions. Kolloid-Z. Z. Polym..

[56] Brostow W., Hagg Lobland H.E., Narkis M. (2006). Sliding wear, viscoelasticity, and brittleness of polymers. J. Mater. Res..

[57] Pothan L.A., Oommen Z., Thomas S. (2003). Dynamic mechanical analysis of banana fiber reinforced polyester composites. Compos. Sci. Technol..

[58] Einstein A., Furth R. (1956). Investigations on the Theory of Brownian Movement.

[59] Barczewski M., Lewandowski K., Rybarczyk D., Kloziński A. (2020). Rheological and single screw extrusion processability studies of isotactic polypropylene composites filled with basalt powder. Polym. Test..

[60] Du J., Wang Y., Xie X., Xu M., Song Y. (2017). Styrene-Assisted Maleic Anhydride Grafted Poly(lactic acid) as an Effective Compatibilizer for Wood Flour/Poly(lactic acid) Bio-Composites. Polymers.

[61] Carreau P.J., DeKee D.C.R., Chhabra R.P. (1997). Rheology of Polymeric Systems.

[62] Li Y., Han C., Bian J., Han L., Dong L., Gao G. (2012). Rheology and biodegradation of polylactide/silica nanocomposites. Polym. Compos..

[63] Gu S.-Y., Zou C.-Y., Zhou K., Ren J. (2009). Structure-rheology responses of polylactide/calcium carbonate composites. J. Appl. Polym. Sci..

[64] Yang J., Nie S., Qiao Y., Liu Y., Li Z., Cheng G. (2019). Crystallization and Rheological Properties of the Eco-friendly Composites Based on Poly (lactic acid) and Precipitated Barium Sulfate. J. Polym. Environ..

[65] Stabik J. (2004). Selected Problems of Rheology of Plasticized Filled Polymers.

[66] Acik E., Orbey N., Yilmazer U. (2016). Rheological properties of poly(lactic acid) based nanocomposites: Effects of different organoclay modifiers and compatibilizers. J. Appl. Polym. Sci..

[67] Liu L., Wang F., Xue P., Wang S. (2019). Influence of interfacial condition on rheological instability behavior of UHMWPE/HDPE/nano-SiO2 blends in capillary extrusion. Rheol. Acta.

[68] Aho J. (2011). Rheological Characterization of Polymer Melts in Shear and Extension: Measurement Reliability and Data for Practical Processing. Ph.D. Thesis.

[69] Andrzejewski J., Skórczewska K., Kloziński A. (2020). Improving the Toughness and Thermal Resistance of Polyoxymethylene/Poly(lactic acid) Blends: Evaluation of Structure–Properties Correlation for Reactive Processing. Polymers.

[70] Su S., Duhme M., Kopitzky R. (2020). Uncompatibilized PBAT/PLA Blends: Manufacturability, Miscibility and Properties. Materials.

[71] Aid S., Eddhahak A., Ortega Z., Froelich D., Tcharkhtchi A. (2017). Experimental study of the miscibility of ABS/PC polymer blends and investigation of the processing effect. J. Appl. Polym. Sci..

[72] Hoseini M., Haghtalab A., Famili M.H.N. (2017). Rheology and morphology study of immiscible linear low-density polyethylene/poly(lactic acid) blends filled with nanosilica particles. J. Appl. Polym. Sci..

[73] Van Gurp M., Palmen J. (1998). Time temperature superposition for polymeric blends. Rheol. Bull..

[74] Gupta A., Simmons W., Schueneman G.T., Hylton D., Mintz E.A. (2017). Rheological and Thermo-Mechanical Properties of Poly(lactic acid)/Lignin-Coated Cellulose Nanocrystal Composites. ACS Sustain. Chem. Eng..

[75] Zhou Z., Zhang Y., Zhang Y., Yin N. (2008). Rheological behavior of polypropylene/octavinyl polyhedral oligomeric silsesquioxane composites. J. Polym. Sci. Part B Polym. Phys..

[76] Pionteck J., Melchor Valdez E.M., Piana F., Omastová M., Luyt A.S., Voit B. (2015). Reduced percolation concentration in polypropylene/expanded graphite composites: Effect of viscosity and polypyrrole. J. Appl. Polym. Sci..

[77] Tran T.P.T., Bénézet J.-C., Bergeret A. (2014). Rice and Einkorn wheat husks reinforced poly(lactic acid) (PLA) biocomposites: Effects of alkaline and silane surface treatments of husks. Ind. Crops Prod..

[78] Silva A.L.N., Cipriano T.F., da Silva A.H.M.d.F.T., Rocha M.C.C.G., Sousa A.F., da Silva G.M. (2014). Thermal, rheological and morphological properties of poly (lactic acid) (PLA) and talc composites. Polímeros Ciência Tecnol..

[79] Nagarajan V., Mohanty A.K., Misra M. (2016). Crystallization behavior and morphology of polylactic acid (PLA) with aromatic sulfonate derivative. J. Appl. Polym. Sci..

[80] Scaffaro R., Maio A., Gulino E.F., Megna B. (2019). Structure-property relationship of PLA-Opuntia Ficus Indica biocomposites. Compos. Part B Eng..

[81] Barczewski M., Sałasińska K., Kloziński A., Skórczewska K., Szulc J., Piasecki A. (2019). Application of the Basalt Powder as a Filler for Polypropylene Composites with Improved Thermo-Mechanical Stability and Reduced Flammability. Polym. Eng. Sci..

[82] Drieskens M., Peeters R., Mullens J., Franco D., Lemstra P.J., Hristova-Bogaerds D.G. (2009). Structure Versus Properties Relationship of Poly(lactic acid). I. Effect of Crystallinity on Barrier Properties. J. Polym. Sci. Part B Polym. Phys..

[83] Andrzejewski J., Krawczak A., Wesoły K., Szostak M. (2020). Rotational molding of biocomposites with addition of buckwheat husk filler. Structure-property correlation assessment for materials based on polyethylene (PE) and poly(lactic acid) PLA. Compos. Part B Eng..

[84] Wang L., Wang Y., Huang Z., Weng Y. (2015). Heat resistance, crystallization behavior, and mechanical properties of polylactide/nucleating agent composites. Mater. Des..

[85] Moraczewski K., Stepczyńska M., Malinowski R., Budner B., Karasiewicz T., Jagodziński B. (2018). Selected properties of polylactide containing natural antiaging compounds. Polym. Adv. Technol..

[86] Zaaba N.F., Ismail H. (2019). A Review on Peanut Shell Powder Reinforced Polymer Composites. Polym. Technol. Mater..

[87] Bulanda K., Oleksy M., Oliwa R., Budzik G., Gontarz M. (2020). Biodegradable polymer composites based on polylactide used in selected 3D technologies. Polimery.

[88] Ashori A., Kiani H., Mozaffari S.A. (2011). Mechanical properties of reinforced polyvinyl chloride composites: Effect of filler form and content. J. Appl. Polym. Sci..

[89] Liu G., Zhang X., Wang D. (2014). Tailoring Crystallization: Towards High-Performance Poly(lactic acid). Adv. Mater..

[90] Fiore V., Di Bella G., Valenza A. (2011). Glass-basalt/epoxy hybrid composites for marine applications. Mater. Des..

[91] Kulinski Z., Piorkowska E. (2005). Crystallization, structure and properties of plasticized poly(l-lactide). Polymer.

[92] Quiles-Carrillo L., Montanes N., Sammon C., Balart R., Torres-Giner S. (2018). Compatibilization of highly sustainable polylactide/almond shell flour composites by reactive extrusion with maleinized linseed oil. Ind. Crops Prod..

[93] Czarnecka-Komorowska D., Mencel K. (2014). Modification of polyamide 6 and polyoxymethylene with [3-(2-aminoethyl)amino]propyl-heptaisobutylpolysilsesquioxane nanoparticles. Przem. Chem..

[94] Chun K.S., Husseinsyah S. (2014). Polylactic acid/corn cob eco-composites. J. Thermoplast. Compos. Mater..

[95] Jandas P.J., Mohanty S., Nayak S.K., Srivastava H. (2011). Effect of surface treatments of banana fiber on mechanical, thermal, and biodegradability properties of PLA/banana fiber biocomposites. Polym. Compos..

[96] Singha K. (2012). A Short Review on Basalt Fiber. Int. J. Text. Sci..

[97] Pardo S.G., Bernal C., Ares A., Abad M.J., Cano J. (2010). Rheological, thermal and mechanical characterization of fly ash-thermoplastic composites with different coupling agents. Polym. Compos..

[98] Nishitani Y., Kajiyama T., Yamanaka T. (2017). Effect of Silane Coupling Agent on Tribological Properties of Hemp Fiber-Reinforced Plant-Derived Polyamide 1010 Biomass Composites. Materials.

[99] Murariu M., Da Silva Ferreira A., Degée P., Alexandre M., Dubois P. (2007). Polylactide compositions. Part 1: Effect of filler content and size on mechanical properties of PLA/calcium sulfate composites. Polymer.

[100] Simmons H., Tiwary P., Colwell J.E., Kontopoulou M. (2019). Improvements in the crystallinity and mechanical properties of PLA by nucleation and annealing. Polym. Degrad. Stab..

[101] Schmidt S.C., Hillmyer M.A. (2001). Polylactide stereocomplex crystallites as nucleating agents for isotactic polylactide. J. Polym. Sci. Part B Polym. Phys..

[102] Yang B., Wang D., Chen F., Su L.-F., Miao J.-B., Chen P., Qian J.-S., Xia R., Liu J.-W. (2019). Melting and Crystallization Behaviors of Poly(Lactic Acid) Modified with Graphene Acting as a Nucleating Agent. J. Macromol. Sci. Part B.

[103] Wu D., Wu L., Wu L., Xu B., Zhang Y., Zhang M. (2007). Nonisothermal cold crystallization behavior and kinetics of polylactide/clay nanocomposites. J. Polym. Sci. Part B Polym. Phys..

[104] Galeja M., Hejna A., Kosmela P., Kulawik A. (2020). Static and Dynamic Mechanical Properties of 3D Printed ABS as a Function of Raster Angle. Materials.

